# Therapeutic Myths in Solid Organ Transplantation Infectious Diseases

**DOI:** 10.1093/ofid/ofae342

**Published:** 2024-06-15

**Authors:** Kellie J Goodlet, Erin K McCreary, Michael D Nailor, Darina Barnes, Marissa M Brokhof, Sarah Bova, Evan Clemens, Beth Kelly, Alicia Lichvar, Dawn M Pluckrose, Bryant B Summers, Kristen R Szempruch, Stephanie Tchen

**Affiliations:** Department of Pharmacy Practice, Midwestern University, Glendale, Arizona, USA; Department of Medicine, Division of Infectious Diseases, University of Pittsburgh Medical Center, Pittsburgh, Pennsylvania, USA; Department of Pharmacy Services, St Joseph's Hospital and Medical Center, Phoenix, Arizona, USA; Department of Pharmacy, Comprehensive Transplant Center, Cedars Sinai Medical Center, Los Angeles, California, USA; Department of Pharmacy, Rush University Medical Center, Chicago, Illinois, USA; Department of Pharmacy, University of Maryland Medical Center, Baltimore, Maryland, USA; Department of Pharmacy, University of Washington Medical Center, Seattle, Washington, USA; Department of Pharmacy, Indiana University Health, Indianapolis, Indiana, USA; Center for Transplantation, UC San Diego Health, San Diego, California, USA; Department of Pharmacy, Tufts Medical Center, Boston, Massachusetts, USA; Comprehensive Transplant Center, The Ohio State University Wexner Medical Center, Columbus, Ohio, USA; Department of Pharmacy, University of North Carolina Medical Center, Chapel Hill, North Carolina, USA; Department of Pharmacy, Froedtert Hospital, Milwaukee, Wisconsin, USA

**Keywords:** asymptomatic bacteriuria, BK virus, dental prophylaxis, ganciclovir, trimethoprim-sulfamethoxazole, vaccination

## Abstract

Infection management in solid organ transplantation poses unique challenges, with a diverse array of potential pathogens and associated antimicrobial therapies. With limited high-quality randomized clinical trials to direct optimal care, therapeutic “myths” may propagate and contribute to suboptimal or excessive antimicrobial use. We discuss 6 therapeutic myths with particular relevance to solid organ transplantation and provide recommendations for infectious diseases clinicians involved in the care of this high-risk population.

## Teaser

Solid organ transplant recipients have increased vulnerability both to infection-related mortality and harms secondary to intemperate antimicrobial use. This article explores the evidence debunking common myths in transplant infectious diseases and provides guidance to clinicians caring for this unique population.

## Graphical Abstract

**Graphical Abstract:**
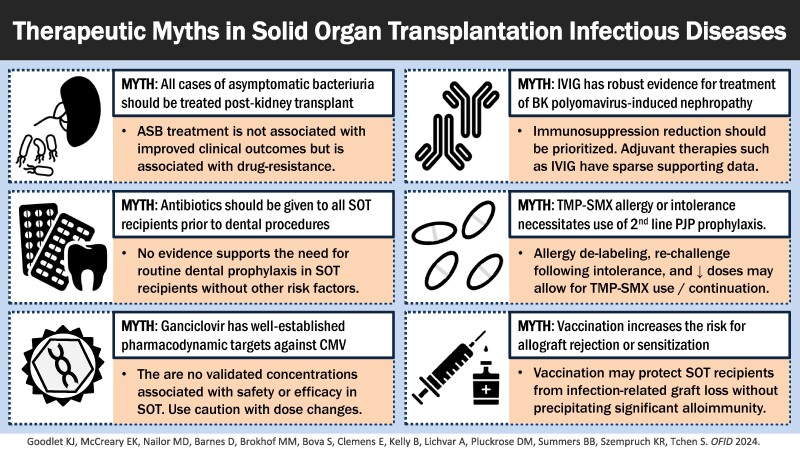

Therapeutics myths are medical beliefs and practices reflecting ostensibly historic data that, upon deeper exploration and review of the literature, lack robust foundational evidence or have been misinterpreted and propagated. Assimilation of myths into one's practice may occur as the result of these lessons being carried forward from early clinical training or unfounded assumptions of the benefits and risks of a particular intervention. In caring for patients at risk for or diagnosed with infectious diseases, perpetuation of myths may lead to selection of inferior antimicrobial agents or receipt of unnecessary antimicrobials. Recent articles have been published debunking some of the most frequently encountered myths relating to the diagnosis and treatment of infectious diseases [1, 2]. Management of infectious diseases in the solid organ transplant (SOT) population poses unique challenges with a broader array of potential pathogens and associated antimicrobial therapies, in addition to limited evidence. Additionally, evolution in immunosuppression regimens and the overall complexity of these patients may make it difficult to maintain current expertise. Clinicians with training in infectious diseases are often consulted by and collaborate with a wide array of medical colleagues involved in the care of the transplant recipient, and they can assist in guiding appropriate antimicrobial selection not only on the individual patient level but also in crafting institutional transplant order sets and protocols relating to infection prophylaxis and treatment. Within this context, the authors present a series of infectious diseases myths with specific relevance to SOT recipients and provide recommendations for optimizing care.

##  

### MYTH 1: ALL CASES OF ASYMPTOMATIC BACTERIURIA SHOULD BE TREATED IN KIDNEY TRANSPLANT RECIPIENTS

Urinary tract infections (UTIs) are the most common infectious complication among kidney transplant recipients (KTRs), occurring most frequently within 6 months posttransplantation [3]. UTIs have been associated with deleterious outcomes, including allograft dysfunction and loss, hospitalization, and mortality, though these results are not observed consistently across all studies [4]. Asymptomatic bacteriuria (ASB), defined within the American Society of Transplantation (AST) 2019 UTI guidelines as the presence of >10^5^ colony-forming units/mL of uropathogen with no urinary or systemic symptoms of infection [5], has been often conflated with UTI or viewed as a harbinger for progression to pyelonephritis and sepsis.

In the first report of infectious complications among KTRs, 9 of 30 recipients with indwelling urethral catheters developed posttransplant bacteriuria; however, it was not specified if this was due to UTI or ASB. Despite no observed association of bacteriuria with mortality, the authors stated: “The long-term effects of bacteriuria following renal homotransplantation have yet to be assessed. It would seem reasonable to assume, however, that lower [UTI] in these patients with a single homograft kidney … could lead to ascending pyelonephritis” [6]. This propagated decades of treatment of any positive urine culture in KTRs, even as antimicrobial and immunosuppressive therapies advanced. However, evidence supporting an association of ASB with increased UTI risk is poor, and ASB treatment in nontransplant populations has been linked to higher, rather than lower, rates of subsequent UTI development [7].

The AST guidelines recommend against routine ASB screening or treatment in KTRs ≥2 months posttransplant. In the setting of persistent ASB (≥2 positive urine cultures with the same organism collected with minimal risk of contamination) within 2 months posttransplant, targeted antibiotic therapy may be considered, although initiation of antibiotics in the absence of infection signs and symptoms may have no benefit and lead to antimicrobial resistance [5]. The 2019 Infectious Diseases Society of America (IDSA) ASB guidelines similarly concluded that, based on the preponderance of evidence, ASB screening and treatment in KTRs after 1 month posttransplant is not warranted. They provided no recommendation for or against ASB treatment within the first month posttransplant due to lack of data [8].

Supporting literature referenced in the 2019 AST and IDSA guidelines comprised mainly retrospective evaluations and included only 2 prospective studies [9]. However, since 2019, additional prospective literature has been published that further corroborates the guideline recommendations against ASB screening and treatment ≥1–2 months posttransplant.

In a multicenter randomized trial of 87 KTRs with ASB ≥1 month posttransplant, no difference in acute graft pyelonephritis was observed at 1 year between KTRs receiving and not receiving antibiotic treatment (risk ratio, 1.40 [95% confidence interval {CI}, .40–4.87]; *P* = .59). No significant differences in graft rejection or dysfunction, hospitalization, or mortality were observed, while antibiotic resistance was more common with ASB treatment [10]. In a larger multicenter, randomized trial of 199 KTRs with ASB ≥2 months posttransplant, antibiotic treatment of ASB for 10 days did not reduce the occurrence of symptomatic UTI compared to no antibiotics (hazard ratio [HR], 0.83 [95% CI, .5–1.4]; *P* = .49). Furthermore, among KTRs with subsequent bacteriuria, those in the antibiotic therapy group were more likely to isolate bacteria resistant to clinically relevant antibiotics compared to the no-therapy group (18% vs 4%; *P* = .003) [11]. While the antibiotics used for treatment in this randomized controlled trial (RCT) were per provider discretion and treatment durations exceeded current standard of care for most cases of UTI, these results still provide robust evidence that antibiotic treatment of ASB after 2 months posttransplant provides no additional benefit to KTRs. Similarly, receipt of antibiotics for ASB ≤2 months post–kidney transplant was not associated with reduced UTI or pyelonephritis risk compared to no antibiotics in a single-center randomized trial of 80 KTRs (HR, 2.8 [95% CI, .8–9.1], *P* = .07 and HR, 6.5 [95% CI, .8–54.7], *P* = .08, respectively) [12]. In both studies cited above, post–ASB guidelines, there was insufficient evidence to examine 2 subsets of populations for potential harms or benefits of treatment, namely patients with retained urological devices (particularly ureteric stents and nephrostomy tubes) and patients with severe neutropenia.

In summary, ASB treatment is not associated with improved clinical outcomes posttransplant but is associated with the emergence of drug-resistant uropathogens. Ceasing routine urine cultures solely for the purpose of bacteriuria screening and associated ASB treatment in KTRs ≥1 month posttransplant is a recommended practice for transplant centers to decrease unnecessary laboratory tests and antibiotic use. Future studies are required to better delineate if ASB treatment may have a role in the setting of severe neutropenia or retained urological devices in the early posttransplant period.

### MYTH 2: ALL SOT RECIPIENTS REQUIRE PROPHYLACTIC ANTIBIOTICS PRIOR TO ROUTINE DENTAL PROCEDURES

Recommendation of prophylactic antibiotics prior to dental procedures was first introduced in 1955 by the American Heart Association (AHA) due to the risk of oral bacteria transiently entering the bloodstream and a further risk of infective endocarditis (IE) whenever disruptions to the oral mucosa occur [13]. This recommendation led to historic widespread use of antibiotic prophylaxis prior to dental procedures, including by >80% of transplant centers [14].

However, in 2007 [15] (and reaffirmed in 2021 [16]), the AHA updated its guidelines to limit prophylaxis only to patients at highest risk for IE, such as those with valvular disease or other structural cardiac defects associated with turbulent blood flow. SOT, in itself, is not an indication for IE prophylaxis, although heart transplant recipients who develop valvulopathy posttransplant are at increased risk both for IE and poor IE outcomes, with mortality as high as 80% [17]. Thus, the AHA recommends that these recipients (heart transplant plus valvulopathy posttransplant) receive antibiotic prophylaxis when an invasive dental procedure is performed, which notably excludes routine teeth cleaning.

The European Society of Cardiology states that antibiotic prophylaxis “may be considered” for all heart transplant recipients while noting a low level of evidence [18]. However, most cases of IE in the SOT population, including heart transplant, are healthcare related and caused by nosocomial pathogens such as *Staphylococcus aureus* rather than oral streptococci, limiting the potential value of dental prophylaxis in IE prevention [19, 20].

Currently, there are no RCTs evaluating the use of antibiotic prophylaxis for dental procedures. This lack of robust supporting data [21] has led to the question of whether antibiotic dental prophylaxis is truly effective for IE prevention even among high-risk patients. Data specific to SOT are even more scarce and do not provide compelling evidence supporting reduced risk of infectious complications. In a retrospective study of KTRs undergoing 190 dental extractions, 56.3% received antibiotic prophylaxis despite only 12 of the 87 unique recipients (13.8%) having antibiotic prophylaxis recommended by their physician. Only 3 recipients (3.4%) had documented cardiac comorbidities. No infectious postoperative complications were identified [22]. Another retrospective study of 65 SOT recipients undergoing dental procedures found that 66.7% were prescribed antibiotic prophylaxis although only 11 (25.6%) had an AHA predisposing cardiac condition. No infectious complications were reported, while 1 patient developed an antibiotic hypersensitivity reaction, demonstrating that antibiotics in this setting caused, rather than prevented, harm [23]. Although not reported in the aforementioned studies, other adverse effects in addition to hypersensitivity to consider when giving antibiotic prophylaxis and acknowledged by the AHA guidelines include the risk of *Clostridioides difficile* infection, drug-related side effects such as QTc prolongation, and selection for antibiotic resistance [16].

Based on available evidence, dental antimicrobial prophylaxis is overprescribed in SOT. This may be attributed in some cases to dental practices rather than transplant team recommendations, highlighting the potential benefits of antimicrobial stewardship in this setting and involving dental practitioners in decisions affecting the care of transplant recipients [24]. While nonemergency dental procedures should be performed before transplantation whenever possible or deferred for 3 months posttransplant in the setting of increased immunosuppression [25], ultimately there is no evidence supporting the need for routine use of dental prophylaxis in all SOT recipients and no evidence for dental prophylaxis for routine cleanings.

### MYTH 3: (VAL)GANCICLOVIR DOSING IS BASED ON DRUG CONCENTRATIONS WITH ESTABLISHED SAFETY AND EFFICACY THRESHOLDS AGAINST CYTOMEGALOVIRUS

In the management of cytomegalovirus (CMV) infection, questions often arise regarding optimal ganciclovir or valganciclovir dosing to eradicate CMV and minimize myelosuppression. Unfortunately, a clear dose-response relationship for efficacy or safety has not been identified for ganciclovir, nor a specific pharmacokinetic-pharmacodynamic target.

Ganciclovir was first synthesized and described as having in vitro human herpesvirus activity in 1983 [26]. An initial dosing strategy of 5 mg/kg intravenously 2–3 times daily was used among 26 patients with severe and life-threatening CMV via a compassionate use pathway; dosing was based on exposures achieved in murine models [27]. In 1988, 314 immunocompromised patients with CMV disease received treatment with either oral ganciclovir solution or intravenous infusion [28]. Initial dosing strategies varied, but induction was typically 5 mg/kg twice daily or 2.5 mg/kg 3 times daily, whereas maintenance doses ranged from 6 mg/kg daily to 5 mg/kg twice weekly. The authors found no relationship between ganciclovir dose and either virologic response or neutropenia; however, they reported lower rates of both neutropenia and clinical response in a prior study among patients with human immunodeficiency virus (HIV) who were treated with 3 mg/kg/day compared to 7.5 mg/kg/day [29]. In the first prospective clinical trial of ganciclovir in SOT recipients, a dose of 5 mg/kg twice daily was utilized (informed by the “induction” dose for patients with AIDS) and has since represented standard dosing for CMV treatment [30]. Valganciclovir 900 mg once daily was first described as producing drug exposures equivalent to 5 mg/kg intravenous ganciclovir; hence, a dosing strategy of 900 mg orally twice daily moved forward for treatment [31].

Despite some laboratories offering a ganciclovir assay for therapeutic drug monitoring (TDM), no established safety or efficacy threshold exists, and robust human pharmacokinetic/pharmacodynamic models are lacking. Wiltshire and colleagues conducted a randomized, double-blind study evaluating exposures of oral ganciclovir versus valganciclovir used for CMV prophylaxis in 364 high-risk SOT recipients [32]. Robust pharmacokinetic sampling was obtained to study the correlation between area under the curve (AUC)_0–24h_ of ganciclovir during prophylaxis and incidence of CMV viremia, disease, and hematological toxicity. Recipients who achieved a ganciclovir AUC >45 µg × hour/mL (more common in recipients receiving valganciclovir compared to oral ganciclovir) had a very low rate of CMV viremia while on prophylaxis and up to 1 month after cessation; however, there was no difference in development of CMV viremia within 6 months or CMV disease within 1 year posttransplantation. The authors noted a weak association between increased ganciclovir exposure and hematological toxicities, but no defined AUC threshold for toxicity. Ritchie and colleagues attempted to establish a relationship between ganciclovir TDM and clinical efficacy and safety by evaluating 82 patients being treated for CMV infection between 2005 and 2015 [33]. The authors of this study did not comment on the CMV serostatus of patients included in this analysis. No association was observed between efficacy outcomes and ganciclovir trough, nor was there a defined association between ganciclovir trough or peak concentrations and any safety endpoint.

In the absence of a validated pharmacodynamic or toxicodynamic threshold, efforts to mitigate ganciclovir toxicities through dose reduction have produced mixed results. For example, while “capping” valganciclovir prophylaxis doses to a maximum of 450 mg daily (more commonly for SOT recipients who are CMV intermediate-risk) has been associated with reduced leukopenia in some retrospective studies, these benefits have not been consistently replicated, and a possible association with CMV breakthrough and ganciclovir-resistant CMV has also been observed. In one study comparing valganciclovir 450 mg daily for 90 days and 900 mg daily for 180 days in 96 high-risk liver transplant recipients for CMV prevention, the standard-dose group experienced significantly more neutropenia (odds ratio, 12.2 [95% CI, 4.17–35.9]). However, ganciclovir-resistant CMV was observed in the reduced-dose group (2 vs 0 patients) [34]. A study comparing valganciclovir 450 mg daily to 900 mg daily in 179 intermediate-risk KTRs found no difference in rates of CMV reactivation (0% vs 3.4%, respectively, *P* = .179) or neutropenia [35]. A study of 90 high-risk SOT recipients observed a lower incidence of leukopenia with reduced-dose valganciclovir (44% vs 75%, *P* < .01), but with a numerically higher rate of CMV breakthrough infection (13.3% vs 2.2%, *P* = .11) [36]. Similar nonsignificant trends were observed in a study of 103 intermediate-risk lung transplant recipients (mean leukocyte nadir of 3400 vs 2800 cells/μL and CMV viremia of 16.4% vs 8.3% for low and standard valganciclovir doses, respectively) [37]. In a large study of 585 SOT recipients, patients receiving valganciclovir prophylaxis doses below manufacturer recommendations were at significantly increased risk for CMV breakthrough after adjusting for other relevant risk factors [38].

In summary, clinicians should be aware that there are no validated ganciclovir concentrations associated with safety or efficacy in SOT, and there does not appear to be a universally supported dosing regimen that will reliably maintain optimal antiviral activity while avoiding toxicity. Decisions surrounding dose reduction of valganciclovir to improve tolerability or mitigate drug-related costs should be weighed against the lack of clear dosing targets and potential efficacy concerns reported in observational studies; indeed, these decisions remain controversial. In SOT recipients who develop myelotoxicity, reducing antiproliferative doses or colony-stimulating factor support may be considered. Additionally, while beyond the scope of this section, the data emerging for safety and efficacy of a preemptive monitoring strategy for CMV prevention in select SOT populations are compelling [39], as well as the use of letermovir as alternative CMV prophylaxis [40, 41].

### MYTH 4: INTRAVENOUS IMMUNOGLOBULIN HAS ROBUST EVIDENCE FOR TREATMENT OF BK NEPHROPATHY

Reactivation of BK polyomavirus (BKPyV) and development of BKPyV-induced nephropathy may result in irreversible renal graft failure. There are currently no US Food and Drug Administration–approved therapies for BKPyV or BKPyV-induced nephropathy. Guidelines recommend immunosuppression reduction as first-line therapy [42], but concerns for precipitating rejection in the early posttransplant period increase the desire for pharmacologic treatments.

Intravenous immunoglobulin (IVIG) has been used clinically for the management of various infectious diseases, including BKPyV, based on the assumption that enough plasma donors had exposure to the infection in question to achieve adequate pathogen-specific antibody concentrations and facilitate a humoral immune response. Lending plausibility to the use of IVIG for BKPyV treatment, population BKPyV seroprevalence may exceed 90% [43], and IVIG preparations have been found to contain BKPyV-neutralizing antibodies [44]. After early evidence of an in vitro interaction with BKPyV [45], Sener and colleagues administered 2 g/kg IVIG divided over 2–5 days (based on daily fluid status and cardiovascular function) to 8 KTRs with biopsy-proven BKPyV-induced nephropathy in conjunction with 50% immunosuppressant dose reduction. Seven (87.5%) remained off dialysis after a mean follow-up of 15 months and 4 (50%) were negative for serum BKPyV DNA polymerase chain reaction at last follow-up [46].

Collectively, studies of IVIG for BKPyV and BKPyV-induced nephropathy are mostly small, retrospective case series that lack randomization or a control group and are confounded by severity of illness. Additionally, all of these studies combine IVIG with other interventions, most commonly immunosuppression reduction, limiting the ability to conclude that favorable outcomes can be attributed to the addition of IVIG versus immunosuppression reduction alone [46, 47]. Among 50 KTRs with BKPyV-induced nephropathy, adjunctive IVIG was associated with more efficient viremia clearance without significant improvement in graft or overall survival, with 1 case of deep vein thrombosis [48]. No association between IVIG use and viral clearance was observed in a similar population with BKPyV-induced nephropathy [49].

Meta-analytic comparisons are similarly limited by data quality. In a systematic review of 40 studies examining immunosuppression reduction alone versus immunosuppression with adjunctive therapies, there were inadequate data to allow for meaningful comparison to IVIG for the primary outcome of pooled death-censored graft loss [50]. In a recent meta-analysis evaluating the outcome of BKPyV clearance, the efficacy of immunosuppression reduction alone was 68% (95% CI, 58%–77%), with the addition of IVIG resulting in a statistically higher clearance rate than reduction in immunosuppression alone (87% [95% CI, 82%–93%]; *P* < .01) [51]. However, only 5 IVIG studies comprising 117 adult patients were included.

There is yet to be a prospective randomized trial evaluating the addition of IVIG to immunosuppression reduction compared to immunosuppression reduction alone, or evidence supporting that IVIG can avert the need for immunosuppression modification or reduce alloimmunity [52]. More research is needed on which immunologic mechanisms may best promote BKPyV immune control; of note, recipient BKPyV seropositivity does not appear protective against reactivation or progression from viruria to viremia [53]. Higher-quality research is needed to understand IVIG's place in therapy, including product selection, timing of administration, and dose optimization. It is important to acknowledge that giving IVIG is not without risks. IVIG products can pose high out-of-pocket costs to patients and may not be covered by insurance. Although typically well-tolerated, serious adverse effects including renal impairment, thrombosis, arrhythmia, aseptic meningitis, hemolytic anemia, and transfusion-related acute lung injury have been reported [54].

Based on the available evidence, transplant centers utilizing IVIG as adjunctive therapy for BKPyV-induced nephropathy may consider limiting this practice to patients with concomitant conditions such as hypogammaglobulinemia that might also be improved with IVIG use and should only utilize IVIG in addition to reduction in immunosuppression.

### MYTH 5: TRIMETHOPRIM-SULFAMETHOXAZOLE ALLERGY HISTORY OR INTOLERANCE NECESSITATES USE OF ALTERNATIVE PNEUMOCYSTIS PROPHYLAXIS

Trimethoprim-sulfamethoxazole (TMP-SMX) represents the drug of choice for *Pneumocystis jirovecii* pneumonia (PJP) prophylaxis in SOT [55]. Following prior studies demonstrating that TMP-SMX can effectively prevent PJP among patients with HIV or hematologic malignancies, TMP-SMX was investigated for use in SOT. Early studies in kidney, lung, heart, and liver transplant corroborated a protective effect of TMP-SMX, most often at doses of 80/400 mg daily or 160/800 mg 3 times weekly [56–58]. Universal PJP prophylaxis for a duration of ≥6–12 months now represents the standard of care [55], with significant decreases in PJP and PJP-related mortality posttransplant [59]. Although large-scale prospective comparative data in SOT are lacking, a retrospective study including 9 French lung transplant centers observed the lowest rate of PJP breakthrough with TMP-SMX prophylaxis (4%) compared to atovaquone (7%) or pentamidine (29%) [60], which is consistent with studies in non-SOT populations [61, 62]. Further, use of TMP-SMX has the advantage of preventing other infections, including toxoplasmosis (in SOT recipients also requiring prophylaxis against *Toxoplasma gondii* [63]), nocardiosis [64], and symptomatic UTI [65].

Despite its aforementioned benefits, concern for adverse effects to TMP-SMX, including hyperkalemia, acute kidney injury, neutropenia, and allergy, may result in initial use or switch to alternative PJP agents in up to 25%–40% of cases [66–69]. However, adverse effects associated with TMP-SMX prophylaxis are often clinically manageable and may be attenuated with alternate dosing strategies. Trimethoprim inhibits active creatinine secretion to cause a reversible rise in serum creatinine not indicative of acute kidney injury [70–72]. Posttransplant hyperkalemia is multifactorial (eg, impaired kidney function, calcineurin inhibitor use) and often remedied with potassium-lowering agents [73]. Hyperkalemia is dose-dependent and usually occurs with therapeutic trimethoprim doses, which are considerably higher than typical doses used for prophylaxis [74–76]. Hematological toxicities are more commonly associated with the use of higher doses in patients with renal dysfunction [77] and other concomitant myelotoxic medications such as valganciclovir, mycophenolic acid, and mTOR inhibitors.

Importantly, SOT recipients who develop non-hypersensitivity-related TMP-SMX adverse effects are able to be successfully rechallenged in 35%–100% of cases [68, 78, 79]. Lower TMP-SMX doses (eg, 80/400 mg 3 times weekly) have also been shown to improve long-term prophylaxis tolerability while maintaining PJP protection and should be considered prior to permanent TMP-SMX cessation [80–82]. It should also be noted that TMP-SMX alternatives carry their own tolerability concerns. Dapsone use can result in hemolytic anemia and methemoglobinemia, and these reactions may occur even among SOT recipients with normal G6PD levels [83]. Atovaquone adverse reactions include gastrointestinal intolerance, rash, and abnormal liver function tests, while cough and bronchospasm represent the major side effects of aerosolized pentamidine. These agents may also represent less affordable options and are only available in suboptimal dosing formulations (unfavorable liquid and nebulized or intravenous, respectively).

After penicillin, allergy to sulfonamides is the most reported allergy and a common reason for alternate PJP prophylaxis [84]. Clinicians should thoroughly discuss sulfonamide allergies with patients to elicit additional information regarding allergic reactions, timing, and severity. Implementation of procedures to promote allergy delabeling among those without a history of severe cutaneous adverse reactions (eg, Stevens-Johnson syndrome) can also expand the pool of SOT recipients able to receive TMP-SMX. Eligibility criteria for 1-step or 2-step TMP-SMX challenge in patients with sulfa allergy have been published by the American Academy of Allergy, Asthma, and Immunology and the American College of Allergy, Asthma, and Immunology [85]. Among a non-HIV population, 95% and 86% rechallenge success rates were reported following 1-step full dose and 2-step TMP-SMX oral challenge, respectively [86].

In summary, TMP-SMX represents the drug of choice for PJP prophylaxis. Allergy delabeling, TMP-SMX rechallenge following intolerance, and alternative TMP-SMX dosing strategies should be considered prior to switching SOT recipients to second-line prophylaxis agents.

### MYTH 6: VACCINES INCREASE THE RISK FOR ALLOGRAFT REJECTION OR SENSITIZATION

Concern for induction of anti-human leukocyte antigen (HLA) antibodies, including de novo donor-specific antibodies (dnDSA) and associated antibody-mediated rejection or graft loss, continue to surround vaccination conversations in SOT. For example, some vaccines (eg, Twinrix, Zostavax, measles-mumps-rubella) contain protein components of human lung fibroblasts including HLA, and development of dnDSA in 1 lung transplant case was proposed to be related to pretransplant vaccination [87]. Another study reported an association of repeat influenza vaccination with positive anti-HLA class I (14.6% vs 3.8% with single vaccination); however, dnDSA were only found in 5 patients, none of whom experienced rejection [88]. A controlled study of KTRs found that 1 of 41 influenza-vaccinated recipients developed dnDSA with increased calculated panel reactive antibody [89].

These small studies do not establish clear temporal relationships between vaccination and dnDSA or rejection. In a large systematic review and meta-analysis before the coronavirus disease 2019 (COVID-19) pandemic, the absolute risk of rejection in noncontrolled studies was small (107 episodes in 5116 recipients [2.1%]) with no increased rejection risk with vaccination [90]. In a study of 169 SOT recipients receiving influenza vaccine, only 5 (2.9%) developed dnDSA and 5 (2.9%) experienced rejection, with 1 patient experiencing both [91]. Similarly, a review of pneumococcal vaccination found no evidence of rejection or generation of dnDSA [92]. A review of vaccinations in lung transplant recipients identified no reports of rejection while highlighting that respiratory viral infections themselves are a source of chronic allograft dysfunction [93]. Most compelling is a review of claims data for 51 730 KTRs with Medicare, of whom 9678 (18.7%) were vaccinated for influenza within 1 year posttransplantation. Vaccination was associated with a lower risk of subsequent allograft loss (adjusted HR [aHR], 0.77 [95% CI, .69–.85]; *P* < .001) and death (aHR, 0.82 [95% CI, .76–.89]; *P* < .001) [94].

Numerous studies have additionally supported that COVID-19 vaccines may be used safely in SOT recipients without precipitating significant alloimmune responses [95–100]. Conversely, reduction of immunosuppression in the setting of COVID-19 infection leads to high rates of dnDSA (30%), with elevated epitope-mismatched patients having the highest risk [101]. Of 112 KTRs hospitalized for COVID-19, 19 (17%) developed dnDSA, but previous vaccination and remdesivir treatment were protective [102]. A study of KTRs undecided about COVID-19 vaccination reported vaccine safety concerns, specifically harm to their transplant, but most also expressed they would proceed with vaccination with specific recommendations from their care team [103]. This highlights the importance of providing clear and accurate education to SOT recipients about the benefits and risks of vaccination.

Overall, there is a lack of convincing evidence that vaccination leads to rejection or allograft harm. Vaccination may protect SOT recipients from infection-related rejection and graft loss in addition to infection-related mortality and should be encouraged according to transplant team recommendations, informed by consensus guidelines [104] and the Centers for Disease Control and Prevention. Notably, the efficacy of vaccines is often reduced by immunosuppression, and the aforementioned guidelines do recommend completion of recommended vaccine series prior to transplant whenever feasible.

## CONCLUSIONS

Infection represents one of the most frequent causes of posttransplant death, underscoring the importance of risk mitigation and timely diagnosis and treatment. At the same time, antimicrobial stewardship in the SOT population is needed to optimize patient outcomes and prevent consequent harms of intemperate antimicrobial administration. Determining which practices earn the label of “myth” can be a controversial endeavor due to their ubiquitous nature. However, both the dispelling and debate of common myths can help break the cycle of unexamined misconceptions entrenched by experience and support effective clinical decision-making by illuminating the limitations of current evidence and spurring future transplant infectious diseases research.

## Note

***Potential conflicts of interest*.** E. K. M. has served on advisory boards for AbbVie, Merck, Basilea, Shionogi, Melinta, Ferring, Cidara, Entasis, LabSimply, Pfizer, and GSK; received honoraria for conference lectures from GSK, Shionogi, and Pfizer; and currently sits on the data and safety monitoring board for the SNAP Trial. M. D. N. has served on advisory boards for Shionogi and InflaRx. All other authors report no potential conflicts.

## References

[1] Johnson MD, Davis AP, Dyer AP, Jones TM, Spires SS, Ashley ED. Top myths of diagnosis and management of infectious diseases in hospital medicine. Am J Med 2022; 135:828–35. 35367180 10.1016/j.amjmed.2022.03.019

[2] McCreary EK, Johnson MD, Jones TM, et al Antibiotic myths for the infectious diseases clinician. Clin Infect Dis 2023; 77:1120–5. 37310038 10.1093/cid/ciad357

[3] Vidal E, Torre-Cisneros J, Blanes M, et al Bacterial urinary tract infection after solid organ transplantation in the RESITRA cohort. Transpl Infect Dis 2012; 14:595–603. 22650416 10.1111/j.1399-3062.2012.00744.x

[4] Hollyer I, Ison MG. The challenge of urinary tract infections in renal transplant recipients. Transpl Infect Dis 2018; 20:e12828. 29272071 10.1111/tid.12828

[5] Goldman JD, Julian K. Urinary tract infections in solid organ transplant recipients: guidelines from the American Society of Transplantation Infectious Diseases Community of Practice. Clin Transplant 2019; 33:e13507. 30793386 10.1111/ctr.13507

[6] Rifkind D, Marchioro TL, Wassell WR, Starzl TE. Infectious diseases associated with renal homotransplantation. JAMA 1964; 189:397–407. 14162136 10.1001/jama.1964.03070060007001 PMC3058948

[7] Cai T, Mazzoli S, Mondaini N, et al The role of asymptomatic bacteriuria in young women with recurrent urinary tract infections: to treat or not to treat? Clin Infect Dis 2012; 55:771–7. 22677710 10.1093/cid/cis534

[8] Nicolle LE, Gupta K, Bradley SF, et al Clinical practice guideline for the management of asymptomatic bacteriuria: 2019 update by the Infectious Diseases Society of America. Clin Infect Dis 2019; 68:e83–110. 30895288 10.1093/cid/ciy1121

[9] Coussement J, Scemla A, Abramowicz D, Nagler EV, Webster AC. Management of asymptomatic bacteriuria after kidney transplantation: what is the quality of the evidence behind the Infectious Diseases Society of America guidelines? Clin Infect Dis 2020; 70:987–8. 10.1093/cid/ciz503 31190055

[10] Sabé N, Oriol I, Melilli E, et al Antibiotic treatment versus no treatment for asymptomatic bacteriuria in kidney transplant recipients: a multicenter randomized trial. Open Forum Infect Dis 2019; 6:ofz243. 31214630 10.1093/ofid/ofz243 PMC6563942

[11] Coussement J, Kamar N, Matignon M, et al Antibiotics versus no therapy in kidney transplant recipients with asymptomatic bacteriuria (BiRT): a pragmatic, multicentre, randomized, controlled trial. Clin Microbiol Infect 2021; 27:398–405. 32919076 10.1016/j.cmi.2020.09.005

[12] Antonio MEE, Cassandra BGC, Emiliano RJD, et al Treatment of asymptomatic bacteriuria in the first 2 months after kidney transplant: a controlled clinical trial. Transpl Infect Dis 2022; 24:e13934. 35980169 10.1111/tid.13934

[13] Prevention of rheumatic fever and bacterial endocarditis through control of streptococcal infections. Mod Concepts Cardiovasc Dis 1956; 25:365–9. 13387501

[14] Guggenheimer J, Mayher D, Eghtesad B. A survey of dental care protocols among US organ transplant centers. Clin Transplant 2005; 19:15–8. 15659128 10.1111/j.1399-0012.2005.00251.x

[15] Wilson W, Taubert KA, Gewitz M, et al Prevention of infective endocarditis: guidelines from the American Heart Association: a guideline from the American Heart Association Rheumatic Fever, Endocarditis, and Kawasaki Disease Committee, Council on Cardiovascular Disease in the Young, and the Council on Clinical Cardiology, Council on Cardiovascular Surgery and Anesthesia, and the Quality of Care and Outcomes Research Interdisciplinary Working Group. Circulation 2007; 116:1736–54. 17446442 10.1161/CIRCULATIONAHA.106.183095

[16] Wilson WR, Gewitz M, Lockhart PB, et al Prevention of viridans group streptococcal infective endocarditis: a scientific statement from the American Heart Association. Circulation 2021; 143:e963–78. 33853363 10.1161/CIR.0000000000000969

[17] Sherman-Weber S, Axelrod P, Suh B, et al Infective endocarditis following orthotopic heart transplantation: 10 cases and a review of the literature. Transpl Infect Dis 2004; 6:165–70. 15762934 10.1111/j.1399-3062.2004.00074.x

[18] Delgado V, Ajmone Marsan N, de Waha S, et al 2023 ESC guidelines for the management of endocarditis. Eur Heart J 2023; 44:3948–4042. 37622656 10.1093/eurheartj/ehad193

[19] Jordan AM, Tatum R, Ahmad D, et al Infective endocarditis following heart transplantation: a systematic review. Transplant Rev (Orlando) 2022; 36:100672. 34826752 10.1016/j.trre.2021.100672

[20] Martínez-Sellés M, Valerio-Minero M, Fariñas MC, et al Infective endocarditis in patients with solid organ transplantation. A nationwide descriptive study. Eur J Intern Med 2021; 87:59–65. 33685806 10.1016/j.ejim.2021.02.017

[21] Rutherford SJ, Glenny AM, Roberts G, Hooper L, Worthington HV. Antibiotic prophylaxis for preventing bacterial endocarditis following dental procedures. Cochrane Database Syst Rev 2022; 5:CD003813. 35536541 10.1002/14651858.CD003813.pub5 PMC9088886

[22] Caliento R, Sarmento DJS, Kobayashi-Velasco S, de Sá SNC, Shibutani PP, Gallotini M. Clinical outcome of dental procedures among renal transplant recipients. Spec Care Dentist 2018; 38:146–9. 29626362 10.1111/scd.12286

[23] Karacaglar E, Akgun A, Ciftci O, Altiparmak N, Muderrisoglu H, Haberal M. Adequacy of infective endocarditis prophylaxis before dental procedures among solid organ transplant recipients. Saudi J Kidney Dis Transpl 2019; 30:764–8. 31464231 10.4103/1319-2442.265450

[24] Teoh L, Thompson W, Suda K. Antimicrobial stewardship in dental practice. J Am Dent Assoc 2020; 151:589–95. 32718488 10.1016/j.esmoop.2020.04.023

[25] US Department of Health and Human Services (HHS) . Dental management of the organ transplant patient. NIH publication No. 11–6270. Bethesda, MD: HHS; 2011.

[26] Martin JC, Dvorak CA, Smee DF, Matthews TR, Verheyden JP. 9-[(1,3-dihydroxy-2-propoxy)methyl]guanine: a new potent and selective antiherpes agent. J Med Chem 1983; 26:759–61. 6302255 10.1021/jm00359a023

[27] Collaborative DHPG Treatment Study Group . Treatment of serious cytomegalovirus infections with 9-(1,3-dihydroxy-2-propoxymethyl)guanine in patients with AIDS and other immunodeficiencies. N Engl J Med 1986; 314:801–5. 3005861 10.1056/NEJM198603273141301

[28] Buhles WC, Mastre BJ, Tinker AJ, Strand V, Koretz SH. Ganciclovir treatment of life- or sight-threatening cytomegalovirus infection: experience in 314 immunocompromised patients. Rev Infect Dis 1988; 10:S495–506. 2847286 10.1093/clinids/10.supplement_3.s495

[29] Laskin OL, Stahl-Bayliss CM, Kalman CM, Rosecan LR. Use of ganciclovir to treat serious cytomegalovirus infections in patients with AIDS. J Infect Dis 1987; 155:323–7. 3027195 10.1093/infdis/155.2.323

[30] Goodrich JM, Mori M, Gleaves CA, et al Early treatment with ganciclovir to prevent cytomegalovirus disease after allogeneic bone marrow transplantation. N Engl J Med 1991; 325:1601–7. 1658652 10.1056/NEJM199112053252303

[31] Martin DF, Sierra-Madero J, Walmsley S, et al A controlled trial of valganciclovir as induction therapy for cytomegalovirus retinitis. N Engl J Med 2002; 346:1119–26. 11948271 10.1056/NEJMoa011759

[32] Wiltshire H, Paya CV, Pescovitz MD, et al Pharmacodynamics of oral ganciclovir and valganciclovir in solid organ transplant recipients. Transplantation 2005; 79:1477–83. 15940035 10.1097/01.tp.0000164512.99703.ad

[33] Ritchie BM, Barreto JN, Barreto EF, et al Relationship of ganciclovir therapeutic drug monitoring with clinical efficacy and patient safety. Antimicrob Agents Chemother 2019; 63:e01855-18. 30602515 10.1128/AAC.01855-18 PMC6395929

[34] Bixby AL, Fitzgerald L, Park JM, Kaul D, Tischer S. Comparison of standard versus low-dose valganciclovir regimens for cytomegalovirus prophylaxis in high-risk liver transplant recipients. Transpl Infect Dis 2021; 23:e13713. 34428337 10.1111/tid.13713

[35] Shi Y, Lerner AH, Rogers R, et al Low-dose valganciclovir is safe and cost-saving in CMV-seropositive kidney transplant recipients. Prog Transplant 2021; 31:368–76. 34839729 10.1177/15269248211046037

[36] Stevens DR, Sawinski D, Blumberg E, Galanakis N, Bloom RD, Trofe-Clark J. Increased risk of breakthrough infection among cytomegalovirus donor-positive/recipient-negative kidney transplant recipients receiving lower-dose valganciclovir prophylaxis. Transpl Infect Dis 2015; 17:163–73. 25661673 10.1111/tid.12349

[37] Hunt J, Chapple KM, Nasar A, Cherrier L, Walia R. Efficacy of low-dose valganciclovir in CMV R^+^ lung transplant recipients: a retrospective comparative analysis. Multidiscip Respir Med 2021; 16:706. 33569173 10.4081/mrm.2021.706 PMC7868948

[38] Khurana MP, Lodding IP, Mocroft A, et al Risk factors for failure of primary (val)ganciclovir prophylaxis against cytomegalovirus infection and disease in solid organ transplant recipients. Open Forum Infect Dis 2019; 6:ofz215. 31211159 10.1093/ofid/ofz215 PMC6559280

[39] Singh N, Winston DJ, Razonable RR, et al Effect of preemptive therapy vs antiviral prophylaxis on cytomegalovirus disease in seronegative liver transplant recipients with seropositive donors: a randomized clinical trial. JAMA 2020; 323:1378–87. 32286644 10.1001/jama.2020.3138 PMC7157180

[40] Limaye AP, Budde K, Humar A, et al Letermovir vs valganciclovir for prophylaxis of cytomegalovirus in high-risk kidney transplant recipients: a randomized clinical trial. JAMA 2023; 330:33–42. 37279999 10.1001/jama.2023.9106 PMC10245286

[41] Martinez S, Sindu D, Nailor MD, et al Evaluating the efficacy and safety of letermovir compared to valganciclovir for the prevention of human cytomegalovirus disease in adult lung transplant recipients. Transpl Infect Dis 2024; 26:e14279. 38742601 10.1111/tid.14279

[42] Hirsch HH, Randhawa PS. BK polyomavirus in solid organ transplantation—guidelines from the American Society of Transplantation Infectious Diseases Community of Practice. Clin Transplant 2019; 33:e13528. 30859620 10.1111/ctr.13528

[43] Knowles WA, Pipkin P, Andrews N, et al Population-based study of antibody to the human polyomaviruses BKV and JCV and the simian polyomavirus SV40. J Med Virol 2003; 71:115–23. 12858417 10.1002/jmv.10450

[44] Randhawa PS, Schonder K, Shapiro R, Farasati N, Huang Y. Polyomavirus BK neutralizing activity in human immunoglobulin preparations. Transplantation 2010; 89:1462–5. 20568674 10.1097/tp.0b013e3181daaaf1 PMC3075564

[45] Puliyanda D, Radha RK, Amet N, et al IVIG contains antibodies reactive with polyoma BK virus and may represent a therapeutic option for BK nephropathy. Am J Transplant 2003; 3:393.

[46] Sener A, House AA, Jevnikar AM, et al Intravenous immunoglobulin as a treatment for BK virus associated nephropathy: one-year follow-up of renal allograft recipients. Transplantation 2006; 81:117–20. 16421486 10.1097/01.tp.0000181096.14257.c2

[47] Anyaegbu EI, Almond PS, Milligan T, Allen WR, Gharaybeh S, Al-Akash SI. Intravenous immunoglobulin therapy in the treatment of BK viremia and nephropathy in pediatric renal transplant recipients. Pediatr Transplantation 2012; 16:e19–24. 10.1111/j.1399-3046.2010.01384.x 22248251

[48] Kable K, Davies CD, O’connell PJ, Chapman JR, Nankivell BJ. Clearance of BK virus nephropathy by combination antiviral therapy with intravenous immunoglobulin. Transplant Direct 2017; 3:e142. 28405598 10.1097/TXD.0000000000000641 PMC5381735

[49] Wadei HM, Rule AD, Lewin M, et al Kidney transplant function and histological clearance of virus following diagnosis of polyomavirus-associated nephropathy (PVAN). Am J Transplant 2006; 6:1025–32. 16611340 10.1111/j.1600-6143.2006.01296.x

[50] Johnston O, Jaswal D, Gill JS, Doucette S, Fergusson DA, Knoll GA. Treatment of polyomavirus infection in kidney transplant recipients: a systematic review. Transplantation 2010; 89:1057–70. 20090569 10.1097/TP.0b013e3181d0e15e

[51] Zhong C, Chen J, Yan Z, et al Therapeutic strategies against BK polyomavirus infection in kidney transplant recipients: systematic review and meta-analysis. Transpl Immunol 2023; 81:101953. 37931665 10.1016/j.trim.2023.101953

[52] Naef B, Nilsson J, Wuethrich RP, Mueller TF, Schachtner T. Intravenous immunoglobulins do not prove beneficial to reduce alloimmunity among kidney transplant recipients with BKV-associated nephropathy. Transpl Int 2021; 34:1481–93. 33872427 10.1111/tri.13882

[53] Bohl DL, Storch GA, Ryschkewitsch C, et al Donor origin of BK virus in renal transplantation and role of HLA C7 in susceptibility to sustained BK viremia. Am J Transplant 2005; 5:2213–21. 16095500 10.1111/j.1600-6143.2005.01000.x

[54] Guo Y, Tian X, Wang X, Xiao Z. Adverse effects of immunoglobulin therapy. Front Immunol 2018; 9:1299. 29951056 10.3389/fimmu.2018.01299 PMC6008653

[55] Fishman JA, Gans H. *Pneumocystis jiroveci* in solid organ transplantation: guidelines from the American Society of Transplantation Infectious Diseases Community of Practice. Clin Transplant 2019; 33:e13587. 31077616 10.1111/ctr.13587

[56] Kramer MR, Stoehr C, Lewiston NJ, Starnes VA, Theodore J. Trimethoprim-sulfamethoxazole prophylaxis for *Pneumocystis carinii* infections in heart-lung and lung transplantation—how effective and for how long? Transplantation 1992; 53:586–9. 1549851 10.1097/00007890-199203000-00019

[57] Olsen SL, Renlund DG, O’Connell JB, et al Prevention of *Pneumocystis carinii* pneumonia in cardiac transplant recipients by trimethoprim sulfamethoxazole. Transplantation 1993; 56:359–62. 8356591 10.1097/00007890-199308000-00021

[58] Torre-Cisneros J, de la Mata M, Lopez-Cillero P, Sanchez-Guijo P, Miño G, Pera C. Effectiveness of daily low-dose cotrimoxazole prophylaxis for *Pneumocystis carinii* pneumonia in liver transplantation—an open clinical trial. Transplantation 1996; 62:1519–21. 8958285 10.1097/00007890-199611270-00026

[59] Stern A, Green H, Paul M, Vidal L, Leibovici L. Prophylaxis for *Pneumocystis* pneumonia (PCP) in non-HIV immunocompromised patients. Cochrane Database Syst Rev 2014; 2014:CD005590. 17636808 10.1002/14651858.CD005590.pub2

[60] Delbove A, Alami H, Tissot A, et al *Pneumocystis pneumonia* after lung transplantation: a retrospective multicenter study. Respir Med 2020; 169:106019. 32442112 10.1016/j.rmed.2020.106019

[61] Vasconcelles MJ, Bernardo MV, King C, Weller EA, Antin JH. Aerosolized pentamidine as *Pneumocystis* prophylaxis after bone marrow transplantation is inferior to other regimens and is associated with decreased survival and an increased risk of other infections. Biol Blood Marrow Transplant 2000; 6:35–43. 10707997 10.1016/s1083-8791(00)70050-4

[62] Warnock AC, Rimland D. Comparison of trimethoprim-sulfamethoxazole, dapsone, and pentamidine in the prophylaxis of *Pneumocystis carinii* pneumonia. Pharmacotherapy 1996; 16:1030–8. 8947975

[63] Schwartz BS, Mawhorter SD. Parasitic infections in solid organ transplantation. Am J Transplant 2013; 13:280–303. 23465021 10.1111/ajt.12120

[64] Passerini M, Nayfeh T, Yetmar ZA, et al Trimethoprim-sulfamethoxazole significantly reduces the risk of nocardiosis in solid organ transplant recipients: systematic review and individual patient data meta-analysis. Clin Microbiol Infect 2024; 30:170–7. 37865337 10.1016/j.cmi.2023.10.008

[65] Fu W, Barahona M, Harkness T, Cohen E, Reardon D, Yoo PS. Higher risk of urinary tract infections in renal transplant recipients receiving pentamidine versus trimethoprim-sulfamethoxazole (TMP-SMX) for *Pneumocystis* pneumonia prophylaxis. Clin Transplant 2020; 34:e14067. 32810885 10.1111/ctr.14067

[66] Gabardi S, Millen P, Hurwitz S, Martin S, Roberts K, Chandraker A. Atovaquone versus trimethoprim-sulfamethoxazole as *Pneumocystis jirovecii* pneumonia prophylaxis following renal transplantation. Clin Transplant 2012; 26:e184–90. 22487221 10.1111/j.1399-0012.2012.01624.x

[67] Giullian JA, Cavanaugh K, Schaefer H. Lower risk of urinary tract infection with low-dose trimethoprim/sulfamethoxazole compared to dapsone prophylaxis in older renal transplant patients on a rapid steroid-withdrawal immunosuppression regimen. Clin Transplant 2010; 24:636–42. 19925478 10.1111/j.1399-0012.2009.01129.x PMC4489856

[68] McLaughlin MM, Galal A, Richardson CL, et al Switch to atovaquone and subsequent re-challenge with trimethoprim-sulfamethoxazole for *Pneumocystis* prophylaxis in a kidney transplant population. Transpl Infect Dis 2017; 19. 10.1111/tid.12769 28833985

[69] Mitsides N, Greenan K, Green D, et al Complications and outcomes of trimethoprim-sulphamethoxazole as chemoprophylaxis for *Pneumocystis* pneumonia in renal transplant recipients. Nephrology (Carlton) 2014; 19:157–63. 24387294 10.1111/nep.12201

[70] Berglund F, Killander J, Pompeius R. Effect of trimethoprim-sulfamethoxazole on the renal excretion of creatinine in man. J Urol 1975; 114:802–8. 1195454 10.1016/s0022-5347(17)67149-0

[71] Delanaye P, Mariat C, Cavalier E, Maillard N, Krzesinski JM, White CA. Trimethoprim, creatinine and creatinine-based equations. Nephron Clin Pract 2011; 119:c187–93. 21832843 10.1159/000328911

[72] Masters PA, O’Bryan TA, Zurlo J, Miller DQ, Joshi N. Trimethoprim-sulfamethoxazole revisited. Arch Intern Med 2003; 163:402–10. 12588198 10.1001/archinte.163.4.402

[73] Shockey W, Wiegel JJ, Parajuli S, Garg N, Swanson KJ, Mandelbrot DA. Potassium-lowering effects of sodium zirconium cyclosilicate in the early post-transplant period. Clin Transplant 2024; 38:e15156. 37812572 10.1111/ctr.15156

[74] Mori H, Kuroda Y, Imamura S, et al Hyponatremia and/or hyperkalemia in patients treated with the standard dose of trimethoprim-sulfamethoxazole. Intern Med 2003; 42:665–9. 12924488 10.2169/internalmedicine.42.665

[75] Perazella MA . Trimethoprim-induced hyperkalaemia: clinical data, mechanism, prevention and management. Drug Saf 2000; 22:227–36. 10738846 10.2165/00002018-200022030-00006

[76] Velázquez H, Perazella MA, Wright FS, Ellison DH. Renal mechanism of trimethoprim-induced hyperkalemia. Ann Intern Med 1993; 119:296–301. 8328738 10.7326/0003-4819-119-4-199308150-00008

[77] Fishman JA . *Pneumocystis jiroveci*. Semin Respir Crit Care Med 2020; 41:141–57. 32000290 10.1055/s-0039-3399559

[78] Lum J, Echenique I, Athans V, Koval CE. Alternative *Pneumocystis* prophylaxis in solid organ transplant recipients at two large transplant centers. Transpl Infect Dis 2021; 23:e13461. 32894607 10.1111/tid.13461

[79] Urbancic KF, Ierino F, Phillips E, Mount PF, Mahony A, Trubiano JA. Taking the challenge: a protocolized approach to optimize *Pneumocystis pneumonia* prophylaxis in renal transplant recipients. Am J Transplant 2018; 18:462–6. 28898546 10.1111/ajt.14498 PMC5790633

[80] Chen RY, Li DW, Wang JY, et al Prophylactic effect of low-dose trimethoprim-sulfamethoxazole for *Pneumocystis jirovecii* pneumonia in adult recipients of kidney transplantation: a real-world data study. Int J Infect Dis 2022; 125:209–15. 36243280 10.1016/j.ijid.2022.10.004

[81] Prasad GVR, Beckley J, Mathur M, et al Safety and efficacy of prophylaxis for *Pneumocystis jirovecii* pneumonia involving trimethoprim-sulfamethoxazole dose reduction in kidney transplantation. BMC Infect Dis 2019; 19:311. 30953458 10.1186/s12879-019-3944-0 PMC6451305

[82] Zmarlicka M, Martin ST, Cardwell SM, Nailor MD. Tolerability of low-dose sulfamethoxazole/trimethoprim for *Pneumocystis jirovecii* pneumonia prophylaxis in kidney transplant recipients. Prog Transplant 2015; 25:210–6. 26308779 10.7182/pit2015153

[83] Lee I, Barton TD, Goral S, et al Complications related to dapsone use for *Pneumocystis jirovecii* pneumonia prophylaxis in solid organ transplant recipients. Am J Transplant 2005; 5:2791–5. 16212642 10.1111/j.1600-6143.2005.01079.x

[84] Zhou L, Dhopeshwarkar N, Blumenthal KG, et al Drug allergies documented in electronic health records of a large healthcare system. Allergy 2016; 71:1305–13. 26970431 10.1111/all.12881 PMC12841114

[85] Khan DA, Banerji A, Blumenthal KG, et al Drug allergy: a 2022 practice parameter update. J Allergy Clin Immunol 2022; 150:1333–93. 36122788 10.1016/j.jaci.2022.08.028

[86] Krantz MS, Stone CA Jr, Abreo A, Phillips EJ. Oral challenge with trimethoprim-sulfamethoxazole in patients with “sulfa” antibiotic allergy. J Allergy Clin Immunol Pract 2020; 8:757–60.e4. 31319222 10.1016/j.jaip.2019.07.003 PMC6960372

[87] Lobashevsky AL, Rosner KM, May JD, et al Rapid and strong de novo donor-specific antibody development in a lung transplant recipient: short communication/case report. Transpl Immunol 2017; 40:17–21. 27979771 10.1016/j.trim.2016.12.003

[88] Cordero E, Bulnes-Ramos A, Aguilar-Guisado M, et al Effect of influenza vaccination inducing antibody mediated rejection in solid organ transplant recipients. Front Immunol 2020; 11:1917. 33123119 10.3389/fimmu.2020.01917 PMC7574595

[89] Khishigsuren B, Demir E, Akgul SU, et al Panel reactive antibody responses against influenza vaccination in kidney transplant recipients. Transplant Proc 2019; 51:1115–7. 31101183 10.1016/j.transproceed.2019.02.009

[90] Mulley WR, Dendle C, Ling JEH, Knight SR. Does vaccination in solid-organ transplant recipients result in adverse immunologic sequelae? A systematic review and meta-analysis. J Heart Lung Transplant 2018; 37:844–52. 29609844 10.1016/j.healun.2018.03.001

[91] Vermeiren P, Aubert V, Sugamele R, et al Influenza vaccination and humoral alloimmunity in solid organ transplant recipients. Transpl Int 2014; 27:903–8. 24797932 10.1111/tri.12345

[92] Dendle C, Stuart RL, Mulley WR, Holdsworth SR. Pneumococcal vaccination in adult solid organ transplant recipients: a review of current evidence. Vaccine 2018; 36:6253–61. 30217523 10.1016/j.vaccine.2018.08.069

[93] Scharringa S, Hoffman T, van Kessel DA, Rijkers GT. Vaccination and their importance for lung transplant recipients in a COVID-19 world. Expert Rev Clin Pharmacol 2021; 14:1413–25. 34328054 10.1080/17512433.2021.1961577

[94] Hurst FP, Lee JJ, Jindal RM, Agodoa LY, Abbott KC. Outcomes associated with influenza vaccination in the first year after kidney transplantation. Clin J Am Soc Nephrol 2011; 6:1192–7. 21511837 10.2215/CJN.05430610 PMC3087788

[95] Al Jurdi A, Gassen RB, Borges TJ, et al Non-invasive monitoring for rejection in kidney transplant recipients after SARS-CoV-2 mRNA vaccination. Front Immunol 2022; 13:838985. 35281011 10.3389/fimmu.2022.838985 PMC8913529

[96] Cassaniti I, Gregorini M, Bergami F, et al Effect of a third dose of SARS-CoV-2 mRNA BNT162b2 vaccine on humoral and cellular responses and serum anti-HLA antibodies in kidney transplant recipients. Vaccines (Basel) 2022; 10:921. 35746528 10.3390/vaccines10060921 PMC9227063

[97] Kueht M, Kirk K, Scott Lea A, et al Donor-directed immunologic safety of COVID-19 vaccination in renal transplant recipients. Hum Immunol 2022; 83:607–12. 35871882 10.1016/j.humimm.2022.07.002 PMC9279300

[98] McCune TR, Bray RA, Baran DA, et al Development of donor-specific antibodies after SARS-CoV-2 vaccination in kidney and heart transplant recipients. Transpl Immunol 2022; 75:101722. 36152939 10.1016/j.trim.2022.101722 PMC9492402

[99] Nishida H, Takai S, Ito H, et al Anti-human leukocyte antigen and anti-ABO antibodies after SARS-CoV-2 mRNA vaccination in kidney transplant recipients. Clin Transplant 2023; 37:e14952. 36846878 10.1111/ctr.14952

[100] Taheri S . Efficacy and safety of booster vaccination against SARS-CoV-2 in dialysis and renal transplant patients: systematic review and meta-analysis. Int Urol Nephrol 2023; 55:791–802. 36723829 10.1007/s11255-023-03471-x PMC9890430

[101] Castrezana-Lopez K, Malchow R, Nilsson J, et al Association between PIRCHE-II scores and de novo allosensitization after reduction of immunosuppression during SARS-CoV-2 infection in kidney transplant recipients. Transpl Infect Dis 2023; 25:e14052. 36884207 10.1111/tid.14052

[102] Killian JT Jr, Houp JA, Burkholder GA, et al COVID-19 vaccination and remdesivir are associated with protection from new or increased levels of donor-specific antibodies among kidney transplant recipients hospitalized with COVID-19. Transpl Int 2022; 35:10626. 35928347 10.3389/ti.2022.10626 PMC9343962

[103] Tharmaraj D, Dendle C, Polkinghorne KR, Mulley WR. Kidney transplant recipients’ attitudes toward COVID-19 vaccination and barriers and enablers to vaccine acceptance. Transpl Infect Dis 2022; 24:e13749. 34694682 10.1111/tid.13749 PMC8646313

[104] Danziger-Isakov L, Kumar D. Vaccination of solid organ transplant candidates and recipients: guidelines from the American Society of Transplantation Infectious Diseases Community of Practice. Clin Transplant 2019; 33:e13563. 31002409 10.1111/ctr.13563

